# Novel Systemic Anticancer Therapy and Healthcare Utilization at the End of Life: A Retrospective Cohort Study

**DOI:** 10.1002/cam4.70450

**Published:** 2024-12-09

**Authors:** Vikas Garg, Alejandra Ruiz Buenrostro, Katrina Heuniken, Rebecca Bagnarol, Mohamed Yousef, Katrina Sajewicz, Suman Dhanju, Kirsten Wentlandt, John Kuruvilla, Stephanie Lheureux, Camilla Zimmermann, Breffni Hannon

**Affiliations:** ^1^ Department of Medical Oncology and Hematology Princess Margaret Cancer Centre, University Health Network Toronto Ontario Canada; ^2^ Division of Medical Oncology, Faculty of Medicine University of Toronto Toronto Ontario Canada; ^3^ Department of Supportive Care University Health Network Toronto Ontario Canada; ^4^ Division of Palliative Medicine, Department of Medicine University of Toronto Toronto Ontario Canada; ^5^ Department of Biostatistics University Health Network Toronto Ontario Canada; ^6^ Temerty Faculty of Medicine University of Toronto Toronto Ontario Canada; ^7^ Regional Cancer Program, Princess Margaret Cancer Centre University Health Network Toronto Ontario Canada; ^8^ Division of Palliative Care, Department of Family and Community Medicine University of Toronto Toronto Ontario Canada; ^9^ Division of Hematology, Faculty of Medicine University of Toronto Toronto Ontario Canada

**Keywords:** end‐of‐life care, healthcare resource utilization, immunotherapy, systemic anticancer therapy

## Abstract

**Background:**

Novel systemic anticancer therapies (SACT) in the form of targeted and immunotherapies are increasingly replacing traditional chemotherapy. Little is known about the impact of novel SACT on healthcare resource utilization (HCRU) at the end of life.

**Methodology:**

A retrospective review of patients attending a tertiary cancer center in Toronto, Canada, with advanced solid or hematological malignancies who died in 2019. Demographic and cancer data, SACT use, HCRU (emergency room [ER] visits, acute/intensive care unit [ICU] admission, and place of death) were retrieved and compared between those who received SACT in their last 30 days of life and those who did not. Chi‐squared tests or Quasi‐Poisson regression calculated HCRU expressed as percentages or rate ratios (RR). Univariate and multivariable logistic regression identified factors independently associated with SACT use.

**Results:**

Of 443 patients included, 88 (20%) received SACT in the last 30 days of life, with 42 (48%) receiving targeted therapies and 10 (11%) immunotherapy. Factors associated with SACT use included younger age (*p* = 0.016), breast (*p* < 0.001), lung (*p* = 0.047), hematological malignancies (*p* = 0.002), fewer comorbidities (*p* = 0.039), and novel SACT (*p* = 0.006). Receipt of SACT was associated with a higher frequency of ER visits (55% vs. 36% who did not receive SACT, *p* = 0.001), acute hospitalizations (68% vs. 47%, *p* < 0.001), ICU admissions (18% vs. 7%, *p* = 0.003), and death in hospital (45% vs. 30%, *p* = 0.008).

**Conclusion:**

Novel SACT use at the end of life is high and is strongly associated with HCRU. Future studies should explore the impact of advance care planning and palliative care referrals on SACT use.

## 1 Introduction

Cancer is the most common cause of death in Canada, contributing to over a quarter of the total annual mortality rate. Projections for 2023 anticipate 239,100 new cancer cases and 86,700 cancer‐related deaths [1]. A considerable portion of these cases are diagnosed at an advanced stage, underscoring the critical role of systemic anticancer therapy (SACT) in their management. The landscape of cancer treatment continues to evolve, witnessing ever‐increasing annual approvals of diverse SACT, including novel targeted therapies and immunotherapies. Many of these therapies have been approved for cases that have relapsed beyond the initial treatment line, emphasizing their predominantly palliative nature [2, 3, 4].

The past several decades have also seen an increasing trend in the use of SACT in the last 30 days of life [5, 6]. Receipt of SACT at the end of life (EOL) has been linked to high rates of healthcare resource utilization (HCRU), defined as acute hospitalizations, admissions to the intensive care units (ICUs), visits to the emergency rooms (ER), and death in hospital, in addition to escalating treatment‐related costs [7, 8, 9, 10, 11]. These interventions have, in turn, been associated with delays in initiating advance care planning discussions, impeding the transition to hospice care, and creating disparities between the desired and actual place of death [12]. This not only compromises the quality of healthcare delivery but also detrimentally affects the health‐related quality of life for patients navigating the complex landscape of advanced cancer [13].

In response to these trends, in 2012 the American Society of Clinical Oncology and the National Quality Forum developed a quality measure with the goal of reducing chemotherapy use within 14 days of death; this predated the advent of many of the therapies currently offered to patients with advanced cancer today, however [14]. While one study suggests that receipt of any type of SACT at EOL is associated with higher rates of acute hospitalizations and healthcare costs, novel SACT agents are often reported to exhibit fewer side effects than conventional chemotherapy treatments [15, 16, 17, 18, 19].

A knowledge gap exists regarding the influence of novel SACT at EOL on healthcare resource utilization (HCRU). This study aimed to evaluate SACT utilization during the last 30 days of life among patients treated at a tertiary care cancer center in Canada. Additionally, we aimed to assess the factors influencing SACT use at the EOL and compare HCRU between patients who received SACT in the last 30 days of life and those who did not.

## 2 Materials and Methods

### 2.1 Study Design

For this retrospective cohort study, the Ontario Cancer Registry was used to identify patients with advanced solid and hematological cancers followed by medical oncology or malignant hematology services at the Princess Margaret Cancer Centre (PM) who died in any setting between January 1, 2019, and December 31, 2019. This time‐period was chosen as it was prior to the beginning of the COVID‐19 pandemic when cancer treatments were temporarily disrupted, and to allow for collection of accurate date of death data. Advanced solid tumors were defined as stage IV for all cancer types or stage III for primary tumors associated with a poor prognosis such as upper gastrointestinal (pancreas, gallbladder), or hormone refractory prostate cancers. Advanced hematological malignancies were defined as those treated with third line treatment or beyond. The study was approved by the University Health Network Research Ethics Board.

### 2.2 Cohort Selection and Variable Definitions

Once death dates were confirmed, data pertaining to the last 30 days of life were reviewed and extracted from the electronic patient record. Data extracted included demographic information about age, sex, postal code, preferred language, date of death, place of death, cancer diagnosis, and comorbidities. We also extracted information on the date of diagnosis of advanced cancer, date of last outpatient oncology visit, date of last administered SACT (defined as any cancer treatment administered systemically for advanced cancer, including intravenous and oral cytotoxic chemotherapy, oral targeted therapy, oral or parenteral endocrine therapy, and immunotherapy) [20], name of SACT, mode of delivery of last SACT (oral or parenteral), type of SACT (chemotherapy, targeted therapy, immunotherapy), SACT received in the last 30 days of life, and radiation therapy received in the last 30 days of life. Combination therapies involving chemotherapy and immunotherapy or chemotherapy and targeted therapies were considered under immunotherapy and targeted therapies, respectively. For HCRU, we extracted the following data within the last 30 days of life: number of ER visits, number of acute hospital admissions, length of stay of last hospital admission, ICU admissions, and place of death (hospital, home or other).

### 2.3 Statistical Analysis

Baseline descriptive characteristics for patients were examined, and factors related to patient demographics, cancer diagnoses, and treatment modalities between patients who received SACT in the last 30 days of life were compared with those who did not, using *t*‐tests and chi‐squared tests.

Unadjusted logistic regression analyses were used to examine differences in associations with patient demographic and clinical characteristics between those patients who received SACT in the last 30 days of life and those who did not. Covariates significant on unadjusted regression (*p* < 0.05) and variables known to be associated with the SACT administration were included in the multivariable logistic regression model. All tests were two‐sided, considering *p* < 0.05 to indicate statistical significance.

## 3 Results

### 3.1 Demographic Characteristics

The first 639 patients of 2893 identified in the Ontario Cancer Registry were screened for inclusion, 196 were excluded for the following reasons: cause of death was unrelated to cancer or treatment (*n* = 71); cancer was not active at the time of death (*n* = 31); date of death was outside of study window or unavailable (*n* = 41); cause of death was unknown (*n* = 39); or most oncology care was provided at another center (*n* = 14). The remaining 443 patients were included in the analyses. The study cohort's baseline characteristics are summarized in Table 1. The median age at time of death was 72 years (range 30–102 years), with 41% of patients aged 75 years or older; 54% were male. The distribution of cancer types within the cohort was diverse: 22% had cancers of the gastrointestinal tract, followed by lung (16%), genitourinary cancers (12%), and leukemia (9%).

**TABLE 1:** Baseline demographic characteristics of the study population.

Parameter	Full Sample, *n* = 443 (%)	No SACT, *n* = 355 (%)	Received SACT, *n* = 88 (%)	*p*
Median age at time of death in years (range)	72 (30–102)	74 (30–102)	69 (30–98)	0.004
**Age category** < 65 years 65–75 years ≥ 75 years	115 (26) 147 (33) 181 (41)	85 (24) 113 (32) 157 (44)	30 (34) 34 (39) 24 (27)	0.013
Male sex	239 (54)	192 (54)	47 (53)	1.00
**Place of residence** Urban Rural	346 (80) 85 (20)	281 (81) 64 (19)	65 (76) 21 (24)	0.28
Cancer site group Gastrointestinal tract Lung cancer Genitourinary Leukemia^a^/MDS/MPN^a^ Gynecological cancer Breast cancer Skin cancers Head and neck cancers Lymphoma Myeloma **Central nervous system** Sarcoma Other^b^	97 (22) 69 (16) 51 (12) 41 (9) 39 (9) 24 (5) 23 (5) 22 (5) 21 (5) 19 (4) 19 (4) 13 (3) 5 (1)	87 (25) 54 (15) 41 (12) 29 (8) 35 (10) 11 (3) 19 (5) 21 (6) 15 (4) 13 (4) 16 (5) 11 (3) 3 (1)	10 (11) 15 (17) 10 (11) 12 (14) 4 (5) 13 (15) 4 (5) 1 (1) 6 (7) 6 (7) 3 (3) 2 (2) 2 (2)	< **0.001**
**Comorbidities** 0–2 ≥ 3	140 (31) 303 (69)	103 (29) 252 (71)	37 (42) 51 (58)	0.051
**Palliative care** Early Late None	161 (37) 67 (15) 211 (48)	129 (37) 61 (17) 162 (46)	32 (37) 6 (7) 49 (56)	0.035

Abbreviation: SACT, systemic anticancer therapy.

^a^ Leukemia including acute and chronic leukemias, myelodysplastic syndrome (MDS), myeloproliferative neoplasm (MPN). Acute myeloid leukemia/AML (*n* = 21), MDS (*n* = 7), chronic lymphocytic leukemia/CLL (*n* = 5), MPN (*n* = 4), acute lymphoblastic leukemia/ALL (*n* = 3), chronic myeloid leukemia‐blast crisis/CML‐BC (*n* = 1).

^b^ Endocrine cancer (*n* = 2), unknown primary (*n* = 2), PECOMA (*n* = 1).

### 3.2 Systemic Anticancer Therapy Administration

**FIGURE 1:**
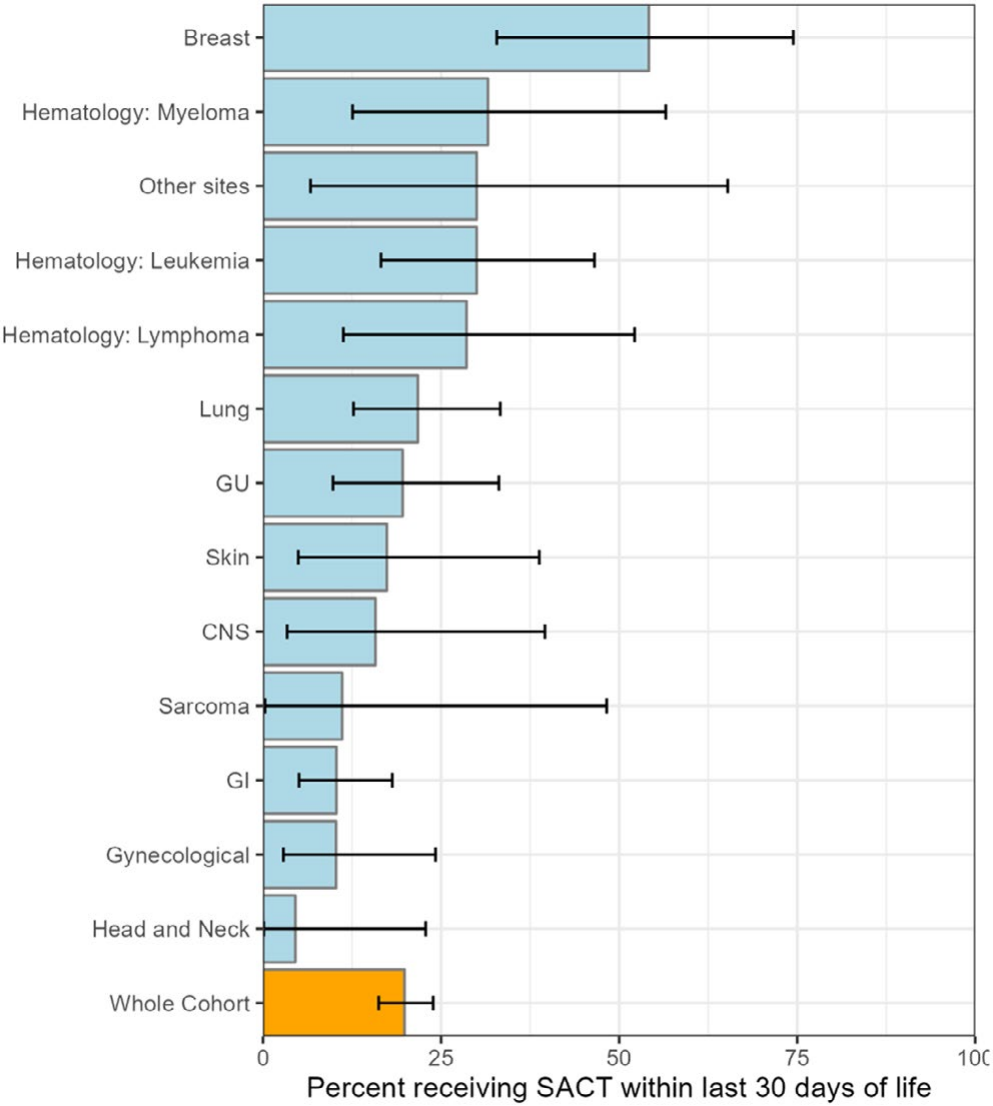
Systemic anticancer therapy (SACT) in the last 30 days of life. Bars represent the proportion of patients in each site group receiving SACT in the last 30 days before death. Error bars represent 95% confidence interval. **Others include sarcoma (*n* = 13), endocrine cancer (*n* = 2), unknown primary (*n* = 2), and PEComa (*n* = 1).

Overall, 305 (69%) patients received SACT at some point following their diagnosis. Of these, 88 (20%) received SACT during the last 30 days of life, with 36 (41%) receiving conventional chemotherapy, 42 (48%) targeted therapies, and 10 (11%) immunotherapeutic agents. Differences in baseline characteristics based on SACT administration during last 30 days of life are provided in Table 1, and details of targeted and immunotherapy agents are presented in Table S1. The median number of lines of SACT received by the patients who received SACT in the last 30 days of life was 2 (range 1–8). The majority (60%) received SACT via parenteral route. Figure 1 illustrates the breakdown of SACT in the last 30 days by tumor site, with breast cancer showing the highest proportion among solid tumor malignancies at 54.2%, followed by 21.7% for lung cancer and 19.6% genitourinary cancers. Patients with hematological malignancies had a high proportion of SACT in the last 30 days of life, with 31.6% patients with multiple myeloma, 30% leukemia and 28.6% lymphoma receiving SACT. Among patients with acute leukemias (acute myeloid leukemia and acute lymphoblastic leukemia) (*n* = 24), the rate of SACT administration in the last 30 days was 41.6% (*n* = 10).

### 3.3 Factors Associated With SACT Administration

Primary factors assessed to explore association with SACT included age at the time of death, cancer site group (malignant hematology vs. solid tumors), number of comorbidities, and duration of cancer diagnosis (see Table 2). Unadjusted and adjusted odds ratios (OR) were calculated to identify factors associated with SACT. Age at the time of death demonstrated a significant association with SACT administration; for every 10‐year increase in age, the odds of receiving SACT decreased (unadjusted OR 0.43, *p* = 0.006; adjusted OR 0.44, *p* = 0.011). Patients with hematological malignancies were more likely to receive SACT compared to those with solid tumors (OR 1.96, *p* = 0.016). Additionally, patients with ≥ 3 comorbidities were less likely to receive SACT compared with those with two or fewer comorbidities (OR 0.56, *p* = 0.019).

**TABLE 2:** Primary factors associated with SACT administration in last 30 days of life.

Parameter	Unadjusted OR (95% CI)	*p* (unadjusted)	Adjusted OR (95% CI)	*p* (adjusted)
Age at time of death^b^		**0.011** ^a^		**0.016** ^a^
< 65 years	Reference		Reference	
65–75 years	0.85 (0.48–1.51)	0.58	0.88 (0.49–1.60)	0.68
≥ 75 years	0.43 (0.24–0.79)	**0.006**	0.44 (0.24–0.83)	**0.011**
Cancer site				
Solid tumors	Reference	**0.016**	Reference	**0.039**
Hematology	1.96 (1.12–3.36)		1.90 (1.03–3.50)	
Number of comorbidities				
0–2	Reference	**0.019**	Reference	**0.039**
≥ 3	0.56 (0.35–0.92)		0.59 (0.35–0.97)	
Duration of cancer^c^	1.00 (0.99–1.00)	0.90	1.00 (0.99–1.00)	0.73

*Note:* Unadjusted/adjusted regression analysis.

Abbreviations: OR, Odds ratio; SACT, Systemic anticancer therapy.

^a^ Global *p* value.

^b^ Age at time of death per 10 years.

^c^ Months from diagnosis to death.

Secondary factors assessed for association with SACT administration at the EOL were type of SACT (chemotherapy, targeted therapy or immunotherapy), mode of SACT delivery (oral vs. parenteral), cancer site group, sex, preferred language, urban versus rural residence (based on postal code), and time from diagnosis to death (Table 3). Of these, statistically significant associations were found for type of SACT (targeted therapy compared with chemotherapy, OR 2.35, *p* = 0.002); hematological malignancies (OR 3.66, *p* = 0.002) and breast cancer (OR 10.28, *p* < 0.001); and mode of SACT delivery (oral route OR 1.76, *p* = 0.033).

**TABLE 3:** Secondary factors associated with SACT administration in last 30 days of life.

Parameter	Unadjusted OR (95% CI)	*p*	*p* (global)
Type of SACT			
Chemotherapy	Reference		
Targeted therapy	2.35 (1.38–4.03)	**0.002**	**0.006**
Immunotherapy	1.73 (0.72–3.94)	0.2	
Mode of delivery			
Parenteral	Reference	**0.033**	
Per oral	1.78 (1.05–2.95)		
Cancer site groups			
Gastrointestinal	Reference		
Genitourinary	2.12 (0.81–5.57)	0.11	**< 0.001**
Gynecological	0.99 (0.26–3.19)	0.99	
Hematology	3.66 (1.67–8.56)	**0.002**	
Lung	2.42 (1.02–5.93)	**0.047**	
Breast	10.28 (3.72–30.03)	**< 0.001**	
Other	1.49 (0.61–3.73)	0.38	
Preferred language			
English	Reference	0.99	
Other	1.01 (0.48–1.98)		
Residence^a^			
Urban	Reference	0.22	
Rural	1.42 (0.80–2.46)		
Sex			
Female	Reference	0.91	
Male	0.97 (0.61–1.56)		

*Note:* Unadjusted Logistic Regression.

^a^ Based on postal code.

### 3.4 Healthcare Resource Utilization (HCRU)

HCRU during the last 30 days of life is summarized in Table 4. In the last 30 days of life, 175 (40%) of patients had one or more ER visits, with a higher percentage among those who received SACT (*n* = 48, 55%) compared to those who did not (*n* = 127, 36%, *p* = 0.002). A similar trend was observed for acute admissions, with 68% of those who received SACT admitted versus 47% who did not (*p* = 0.001). In terms of ICU admissions, 18% of the SACT cohort were admitted to the ICU compared with 7% who did not (*p* = 0.003). While 33% of the overall sample died in hospital, 45% of SACT recipients died in the acute setting versus 30% who did not receive SACT (*p* = 0.008).

**TABLE 4:** Health services utilization in the last 30 days of life.

Parameter	Overall (*n* = 443)	No SACT (*n* = 355)	SACT (*n* = 88)	*p* ^a^
ER visits, *n* (%)				
≥ 1 None	175 (40) 268 (60)	127 (36) 228 (64)	48 (55) 40 (45)	0.002
Acute admissions, *n* (%)				
≥ 1 None	228 (52) 215 (48)	168 (47) 187 (53)	60 (68) 28 (32)	< 0.001
ICU admissions, *n* (%)				
≥ 1 None	41 (9) 402 (91)	25 (7) 330 (93)	16 (18) 72 (82)	0.003

Abbreviations: ER, Emergency room; ICU, intensive care unit; SACT, Systemic anticancer therapy.

^a^ *p*‐values from chi‐squared or Fisher's exact tests.

Compared to chemotherapy or targeted therapies, ER visits (*p* = 0.014), acute admissions (*p* = 0.005), ICU admissions (*p* = 0.003), and in‐hospital deaths (*p* = 0.028) were significantly lower in patients receiving immunotherapies (Table S2). Patients receiving parenteral SACT had a significantly higher number of ER visits (63% vs. 42%, *p* < 0.001), acute admissions (75% vs. 58%, *p* < 0.001), and ICU admissions (25% vs. 8%, *p* = < 0.001) compared to those receiving oral SACT; they were also more likely to die in hospital (50% vs. 39%, *p* = 0.011), Table S3.

Associations between demographic/clinical factors and the mode of delivery of SACT (parenteral vs. oral vs. no SACT) in the last 30 days of life are illustrated in Table S4. Patients over 75 had lower odds of receiving both per‐oral and parenteral SACT than those under 65. While patients aged 65–75 had higher odds of per‐oral SACT (OR = 1.41) and lower odds of parenteral SACT (OR = 0.69) compared to those under 65, neither was statistically significant.

## 4 Discussion

The current study explores the influence of novel SACT during the last 30 days of life on HCRU among patients treated at a tertiary cancer center in Canada. SACT was administered to 20% in the last 30 days of life, predominantly through targeted therapies and conventional chemotherapy. Age, number of comorbidities, and cancer type significantly influenced SACT administration, with older patients, those with a greater number of comorbidities, and patients with solid tumors other than breast and lung cancers, less likely to receive SACT in their last 30 days. Receipt of SACT in the last 30 days of life was associated with statistically significantly higher rates of ER visits, acute admissions, ICU admissions, and hospital deaths compared to patients who did not receive SACT in the last 30 days of life, although, immunotherapy was associated with lower HCRU compared to chemotherapy and targeted therapies. These findings provide insights into the prevalence of traditional and novel SACT utilization at the end of life, factors associated with its administration, and the subsequent impact on HCRU.

SACT administration in the last 30 days of life in our sample (20%) was higher than previously reported studies from Canada, where rates varied between < 5% (last 15 days of life) in a 2011 population‐based study [6] and 17.4% (last 30 days of life) in a study from a regional cancer center between 2016 and 2018 [15]. Cancer registry data from Europe and the United States highlight variable rates of SACT administration during the last 30 days of life, ranging between 4.8% in Norway, 10.6% in the United States, 12.7% in Belgium, 16% in Denmark, and 19.5% in France [21, 22, 23, 24]. In our study, targeted or immunotherapies accounted for 59% SACT at the EOL, compared with only 25.7% in a Canadian study spanning 2016–18 [15]. Overall international trends indicate a reduction in traditional chemotherapy use and an increased use of novel SACT [17, 25]. A US study of patients who died within 4 years of their cancer diagnosis reported that although overall SACT rates within 30 days of death remained constant at 39% in 2015 and 2019, the use of traditional chemotherapy declined from 26% in 2015 to 16% in 2019, while there was an increased use of immunotherapy (5% in 2015; 18% in 2019) [17].

We observed significant associations between SACT administration and several patient and clinical variables, including younger age, fewer comorbidities, specific cancer types (hematologic malignancies and breast cancer), type of SACT (chemotherapy versus targeted therapies), and the mode of SACT delivery. Younger age and fewer comorbidities are consistently reported in the literature as contributing to SACT use at the EOL [6, 22, 23, 24]. Similar to our findings for solid tumors, increased SACT use has been reported for with breast and lung cancer [6], as well as melanoma [15, 22] and testicular cancers [24]. In our cohort, 38.5% of patients with breast cancer received oral hormonal agents which likely contributed to the high utilization of SACT in this subgroup. Our sample included 18% of patients with a hematological malignancy, where high rates of SACT use in the last 30 days of life are frequently reported [6, 23]. This may reflect the acute onset and less predictable disease trajectory associated with some hematological malignancies such as acute leukemia (41.6% of patients with acute leukemia in our cohort received SACT in their last 30 days). Other factors in the literature found to be associated with higher SACT use include race (greater use of SACT among Caucasian patients); private insurance, and in community cancer centers; there is evolving evidence from the US to support the increased use of novel SACT among patients with poor performance status [26]. Our study, from a Canadian academic center, is one of the first to explore these factors in a universal healthcare system setting.

While previous studies have demonstrated an increasing trend in HCRU at the end of life for patients with advanced cancer [21, 27, 28], much of these data predate the novel SACT era dominated by targeted and immunotherapies. Our findings add to the growing evidence around the impact of novel SACT on HCRU at the EOL. In a single‐center retrospective study from the US of patients treated with immunotherapy from 2011 to 2017, 49% of patients had at least one ER visit in the last month of life, 59% underwent at least one hospitalization, and 11% died within the hospital setting [29]. Similarly, high rates of ER visits, acute hospitalizations, ICU admissions and in‐hospital death have been reported among patients receiving immunotherapy in Europe and the US [30, 31, 32]. Our findings align closely with these, with ER visits noted in 55%, acute hospitalization in 69%, and ICU admission in 18% of our SACT cohort. Of note in our study was the high rate of in‐hospital deaths with 33% of the entire cohort and 46% of the SACT group dying in hospital, seemingly at odds with the preferred place of death (home) reported by the majority patients with advanced cancer.

Our study has a number of limitations. The retrospective nature of the study and the focus on a single tertiary care cancer center in an urban setting may limit the generalizability of the findings. We were unable to explore situations where SACT was prescribed with the intention of providing symptom relief in advanced disease. The sample size necessitated grouping of comorbidities and was too small to fit a fully adjusted single model without risking overfitting the data. Uniquely, our center is the largest tertiary cancer facility in Canada, with greater access to clinical trials and treatments that might not be available elsewhere; this may in part have influenced our findings. The study was unable to capture information on several factors that may influence both SACT administration and HCRU at the EOL, including the perspectives of family or caregivers on treatment and EOL decisions, advance care planning discussions and provider decision‐making.

## 5 Future Directions

Future research should explore the influence of palliative care referral and advance care planning discussions on SACT and HCRU use at the EOL. More in‐depth explorations of clinician decision‐making around offering novel SACT, particularly in the context of clinical trials and realistic framing of likely outcomes as well as alternatives to SACT may be illuminating. Interviews with patients and caregivers to better understand factors that influence their decision‐making around accepting or declining SACT as well as patient and caregiver understanding and decision‐making about accepting or declining SACT in advanced disease should be explored. A larger, multi‐site prospective study is essential to address these questions thoroughly.

## 6 Conclusion

We have demonstrated high rates of SACT use and associated HCRU in the last 30 days of life among a cohort of patients with advanced cancer. These data can be used to inform decision‐making about the judicious use of SACT in this patient population.

## Author Contributions

**Vikas Garg:** conceptualization (equal), data curation (equal), formal analysis (equal), methodology (equal), validation (equal), visualization (equal), writing – original draft (lead), writing – review and editing (lead). **Alejandra Ruiz Buenrostro:** conceptualization (equal), data curation (equal), formal analysis (equal), methodology (equal), validation (equal), visualization (equal), writing – original draft (equal), writing – review and editing (equal). **Katrina Heuniken:** formal analysis (equal), visualization (supporting), writing – original draft (supporting), writing – review and editing (supporting). **Rebecca Bagnarol:** data curation (equal), visualization (equal), writing – original draft (equal), writing – review and editing (equal). **Mohamed Yousef:** data curation (equal), visualization (equal), writing – original draft (equal), writing – review and editing (equal). **Katrina Sajewicz:** data curation (equal), visualization (supporting), writing – original draft (supporting), writing – review and editing (supporting). **Suman Dhanju:** conceptualization (equal), funding acquisition (equal), methodology (equal), project administration (equal), resources (supporting), supervision (supporting), writing – review and editing (supporting). **Kirsten Wentlandt:** conceptualization (equal), methodology (equal), project administration (equal), resources (equal), supervision (equal), writing – review and editing (equal). **John Kuruvilla:** conceptualization (equal), funding acquisition (equal), methodology (equal), project administration (equal), resources (equal), supervision (equal), visualization (equal), writing – original draft (equal), writing – review and editing (equal). **Stephanie Lheureux:** conceptualization (equal), funding acquisition (equal), methodology (equal), project administration (equal), resources (equal), supervision (equal), visualization (equal), writing – original draft (equal), writing – review and editing (equal). **Camilla Zimmermann:** conceptualization (equal), funding acquisition (equal), methodology (equal), project administration (equal), resources (equal), supervision (equal), visualization (equal), writing – original draft (equal), writing – review and editing (equal). **Breffni Hannon:** conceptualization (lead), data curation (equal), formal analysis (equal), funding acquisition (equal), methodology (lead), project administration (lead), resources (equal), supervision (lead), validation (equal), visualization (equal), writing – original draft (lead), writing – review and editing (lead).

## Disclosure

J.K.: Research Support from Canadian Cancer Society Research Institute (CCSRI), Canadian Institutes of Health Research (CIHR), Leukemia and Lymphoma Society Canada, Princess Margaret Cancer Foundation, Astra Zeneca, Kite, Merck, Novartis. Honoraria from Abbvie, Amgen, Astra Zeneca, BMS, Beigene, Genmab Gilead, Incyte, Janssen, Merck, Novartis, Pfizer, Roche, and Seattle Genetics. Data Safety Monitoring Board of Karyopharm. C.Z.: PI for a study by Pfizer. No personal honoraria. S.L.: Consulting fees from AstraZeneca, GSK, Merck, Eisai, Roche, Schrodinger and Seagen. She declared honoraria from GSK, AstraZeneca, Roche, Eisai, Merck. Research grants from GSK, AstraZeneca, Roche, Merck, and Repare Therapeutics.

## Ethics Statement

The study was approved by University Health Network Research Ethics Board. Ethics Board granted a waiver for the individual patient consent for this study.

## Conflicts of Interest

The authors declare no conflicts of interest.

## Supporting information


Tables S1–S4.

## Data Availability

The data that support the findings of this study are available on reasonable request from the corresponding author. The data are not publicly available due to privacy or ethical restrictions.
