# Supplementary material for: Novel Systemic Anticancer Therapy and Healthcare Utilization at the End of Life: A Retrospective Cohort Study
Source: Cancer Med. 2024 Dec 9;13(23):e70450. doi: 10.1002/cam4.70450 (PMC11626481; doi:10.1002/cam4.70450)
Supplement: Supplementary file 1 — Tables S1–S4. [file CAM4-13-e70450-s001.docx]

**Table S1. Details of targeted therapy/immunotherapy administered during the last 30 days of life.**

| **Therapeutic agent** | **n (%)** |
| --- | --- |
| **Targeted Therapies**  Tyrosine kinase inhibitor  Monoclonal antibodies*  Hormonal agent  Proteasomal inhibitor**  ADT/ART  mTOR inhibitor  Lenalidomide | **n=42 (%)**  17 (40.5)  11 (26.2)  5 (11.9)  3 (7.1)  3 (7.1)  2 (4.8)  1 (2.4) |
| **Immunotherapy** | **n=10 (%)** |
| Anti PD-1  Anti PD-L1^#^ | 9 (90%)  1 (10%) |

ADT androgen deprivation therapy, ARAT androgen receptor-axis-targeted therapies, mTOR mammalian target of rapamycin, PD-1 programmed cell death protein 1, PD-L1 Programmed Cell Death Ligand 1

* In combination with chemotherapy in 4 patients

** In combination with lenalidomide in 2 patients

# In combination with chemotherapy

**Table S2. Health services utilization in the last 30 days of life based on type of SACT (Chemotherapy, Targeted therapy, Immunotherapy).**

| Parameter | No SACT (n=355) | Chemotherapy (n=36) | Targeted therapy (n=42) | Immunotherapy  (n=10) | p value^#^ |
| --- | --- | --- | --- | --- | --- |
| **ER visits** |  |  |  |  |  |
| Yes | 127 (36) | 20 (56) | 22 (60) | 6 (60) | 0.014 |
| No | 228 (64) | 16 (44) | 20 (48) | 4 (40) |  |
| **Acute admissions** |  |  |  |  |  |
| Yes | 168 (47) | 24 (67) | 30 (71) | 6 (60) | 0.005 |
| No | 187 (53) | 12 (33) | 12 (29) | 4 (40) |  |
| **ICU admissions** |  |  |  |  |  |
| Yes | 25 (7) | 8 (22) | 8 (19) | 0 (0) | 0.003 |
| No | 330 (93) | 28 (78) | 34 (81) | 10 (100) |  |
| **Place of death** |  |  |  |  |  |
| In hospital | 106 (30) | 16 (44) | 20(48) | 4 (40) | 0.041 |
| Not in hospital/ unknown | 249 (70) | 20 (56) | 22 (52) | 6 (60) |  |

^#^ p-values from Chi-squared or Fisher’s exact tests

ER Emergency room, ICU intensive care unit, SACT Systemic anti-cancer therapy

**Table S3. Health services utilization in the last 30 days of life based on mode of delivery of SACT (per oral vs parenteral).**

| Parameter | No SACT (n=355) | Per Oral SACT (n=36) | Parenteral SACT (n=52) | p value^#^ |
| --- | --- | --- | --- | --- |
| **ER visits** |  |  |  |  |
| Yes | 127 (36) | 15 (42) | 33 (63) | <0.001 |
| No | 228 (64) | 21 (58) | 19 (37) |  |
| **Acute admissions** |  |  |  |  |
| Yes | 168 (47) | 21 (58) | 39 (75) | <0.001 |
| No | 187 (53) | 15 (42) | 13 (25) |  |
| **ICU admissions** |  |  |  |  |
| Yes | 25 (7) | 3 (8) | 13 (25) | <0.001 |
| No | 330 (93) | 33 (92) | 39 (75) |  |
| **Place of death** |  |  |  |  |
| In hospital | 106 (30) | 14 (39) | 26 (50) | 0.011 |
| Not in hospital/ unknown | 249 (70) | 22 (61) | 26 (50) |  |

^#^ p-values from Chi-squared or Fisher’s exact tests

ER Emergency room, ICU intensive care unit, SACT Systemic anti-cancer therapy

**Table S4. Associations between demographic factors and mode of delivery of SACT (per oral vs parenteral) in the last 30 days of life.**

| **Variable** | **Category** | **Odds Ratio (95% CI)**  **Per-Oral vs No SACT** | **Odds Ratio (95% CI) Parenteral vs No SACT** |
| --- | --- | --- | --- |
| Age (reference: <65) | 65-75 | 1.413 (0.55-3.60) | 0.694 (0.34-1.39) |
|  | >75 | 0.852 (0.32-2.22) | 0.292 (0.13-0.64) |
| Months from diagnosis  to death | | 1.002 (0.99-1.00) | 0.995 (0.98-1.00) |
| Received Palliative Care (reference: Yes) | No | 1.209 (0.56-2.58) | 1.336 (0.70-2.52) |
| Cancer site (reference: solid-tumour cancers) | Hematological cancers | 2.058 (0.87-4.84) | 1.796 (0.84-3.83) |
| Number of Comorbidities (reference: 0-2) | 3+ | 0.639 (0.30-1.35) | 0.552 (0.29-1.02) |
| Sex (reference: female) | Male | 0.921 (0.44-1.88) | 0.977 (0.53-1.79) |

Multivariable multinomial logistic regression with three possible outcomes: Per-Oral SACT, Parenteral SACT, and no SACT (reference).

Odds ratios can be interpreted as the change in odds of receiving a given SACT (PO or Parenteral) compared to no SACT per unit increase in each explanatory variable.
